# Prominent Alveolar Bone Graft Substitute Derived from Silk Fibroin/Hyaluronic Acid/Demineralized Dentin Matrix Hybrid Hydrogel

**DOI:** 10.34133/bmr.0243

**Published:** 2025-08-19

**Authors:** Runzhi Chen, Wentao Zhang, Yude Ding, Linhong Wang, Yuxin Zheng, Wang Wang, Danni Wu, Zhuoheng Xia, Jing Zhu, Feng Chen, Fan Yang

**Affiliations:** ^1^ Center for Plastic and Reconstructive Surgery, Department of Stomatology, Zhejiang Provincial People’s Hospital, Affiliated People’s Hospital, Hangzhou Medical College, Hangzhou, Zhejiang, China.; ^2^ Hangzhou Geriatric Hospital (Department of Stomatology), Affiliated Hangzhou First People’s Hospital Chengbei Campus, School of Medicine, Westlake University, Hangzhou, Zhejiang, China.; ^3^ Shangyu Tangpu Health Center, Shaoxing, Zhejiang, China.; ^4^ School of Stomatology, Zhejiang Chinese Medical University Hangzhou, Hangzhou, Zhejiang, China.; ^5^ College of Material Science and Engineering, Zhejiang University of Technology, Hangzhou, Zhejiang, China.

## Abstract

Bone graft substitutes are commonly used to repair large bone defect, and restoring the alveolar bone defects in height and width is a major challenge in restorative dentistry. In comparison with clinic bone graft substitutes such as bovine-derived powder and hydroxyapatite, demineralized dentin matrix (DDM) is a valuable alternative due to its compositional similarity to human-derived bone. However, a challenge remains in using DDM for bone rehabilitation, particularly in maintaining spatial morphology due to its granular form. This study developed an effective bone graft substitute using DDM particles in a fast-cured silk fibroin/hyaluronic acid methacrylate (SF/HAMA) hydrogel, which adheres well to the alveolar bone defect and rapidly gels under blue light. In vitro and in vivo experiments were performed to evaluate the biocompatibility of this hybrid hydrogel. The ability to repair bone defects was tested on cranial defects in rats and mandibular defects in beagles. Results showed that the in situ composites exhibited excellent mechanical strength and biocompatibility, with micro-computed tomography and histology confirming the best bone regeneration effect of the SF/HAMA/DDM-50 hybrid hydrogel. This composited bone graft substitute could provide a novel strategy for the clinical treatment of alveolar bone defects and is a promising candidate for bone tissue reconstruction and regeneration.

## Introduction

Bone defects are a challenging clinical problem that can impair mobility and even be life-threatening. Bone defects causes vary, and may include trauma, infection, surgery, or tumor removal. In clinical practice, bone defects are typically repaired using autografts, allografts, and xenografts. These materials are favored for bone regeneration [1,2]. However, each type of bone graft substitute has its limitations. Xenograft, for instance, may be immunogenic [3], and allograft may transmit diseases [4], posing risks of immune rejection and infection [5]. Although autogenous bone is considered the gold standard [6], it resorbs at an uncontrolled rate and requires at least 2 surgeries, which can lead to complications such as nerve damage, infection, and limited donor bone [7].

As early as 1965, dentin was first reported as an osteoinductive material [8]. In 2008, the demineralized dentin matrix (DDM), a type of bone graft substitute obtained by grinding, disinfecting, and demineralizing ex vivo teeth, was first applied clinically [9]. Bone tissue is a complex hierarchical structure, with its main components being hydroxyapatite (HAp), type I collagen fibers, and water (water-soluble organics) [10]. DDM possesses components similar to bone [11], with its primary organic substances being type I collagen fibers and non-collagen proteins. These include proteoglycans (PGs) [12], known as mineralization regulators, and N-linked glycoprotein, a small integrin-binding ligand that plays an important role in bone formation, among others [13]. It also contains various growth factors, such as bone morphogenetic proteins (BMPs) and transforming growth factor-β (TGF-β) [14]. BMP is considered one of the most effective growth factors in inducing bone formation [15]. Histologically, the biological apatite crystals of dentin are deposited on type I collagen protofibers, forming a gradient layered structure, which is similar to that of the bone [16]. During the demineralization process, the growth factors in DDM are released from the matrix due to the loss of bound mineral crystals, endowing DDM with excellent osteoinductive and osteoconductive capabilities [3]. Meanwhile, DDM has low immunogenicity and does not cause severe inflammatory reactions in the host [17], making it an ideal choice for bone graft substitute. However, from a clinical point of view, DDM is considered relatively difficult to operate in bone rehabilitation cases, thus limiting its widespread clinical use. In particular, when repairing large bone defects or requiring vertical bone augmentation of the alveolar bone, maintaining spatial morphology by granular particles of natural DDM is still challenging. The embedded DDM particles could be easily washed out from the implantation site and rapidly dispersed in body fluids, despite the fact that there is the possible excipient such as glycerol or hyaluronic acid (HA). Loose and unformed DDM grafts may not achieve the desired efficacy [18]. Consequently, researchers have increasingly focused on combining it with biocompatible substrates, aiming to improve handling properties while also enhancing bone and dental tissue regeneration [19–22]. Bao et al. [19] demonstrated that the incorporation of fibrin glue with DDM not only improves the physical properties but also enhances osteogenic activities and bone regeneration. Sultan and Jayash revealed that alginate hydrogels can serve as a carrier for DDM, providing good cell compatibility and promoting bone regeneration both in vitro [20] and in vivo [22].

Hydrogels, a 3-dimensional (3D) macromolecule network similar to natural extracellular matrix and high-water content, can provide a platform for cells to transport nutrients, proliferate, and differentiate [23], to meet specific requirements under different conditions, and have great potential in the field of bone tissue regeneration. The goal of developing ideal bone graft substitute that can combine the characteristics of different materials and simulate the microenvironment of bone tissue has become the target of increasing research. It is well known that HA, a glycosaminoglycan primarily found in the connective tissues of vertebrates, is a crucial component of the extracellular matrix [24]. HA exhibits high hydrophilicity, providing tissues with hydroxyl hydration action and active sites for chemical modification [25]. HA participates in various signaling pathways, triggering cellular behavior regulation and promoting bone regeneration [26]. Moreover, HA may be associated with improved osteogenic gene expression. Studies have shown that HA can promote the alkaline phosphatase (ALP) activity, osteogenic-related protein, and mRNA expression in human bone marrow-derived mesenchymal stem cells (hBMSCs) [27]. HA also has immunosuppressive and anti-inflammatory effects, contributing to wound healing and scar prevention [28,29]. However, HA-derived hydrogels have some disadvantages under physical and physiological conditions, such as weak mechanical properties, tardy degradation, and difficulties in forming and fixing, which are particularly inadequate for repairing large bone defects [30]. Hence, there is a need to develop hydrogels with relatively high mechanical strength and biocompatibility to provide new options for the reconstruction and regeneration of bone tissue.

Silk fibroin (SF) is a natural biomacromolecule. It has excellent biocompatibility and biodegradability. Compared to other natural biomacromolecules like gelatin and collagen, SF has superior mechanical properties and weak immunogenicity [31]. The tensile strength and toughness of silk fibers are outstanding, and biological products made from SF have been widely used [32]. To overcome the insufficient mechanical strength and slow gelatinization of HA, we constructed a high-strength hydrogel, which was composed of hyaluronic acid methacrylate (HAMA) and SF modified with glutathione. Methacrylate groups of HAMA polymerized homogeneously in a typical photo-initiated polymerization. They also reacted with mercapto SF to form a cross-linked hydrogel. The gelatinization process was quickly finished in 10 s and blue-light irradiation.

**Fig. 1.**
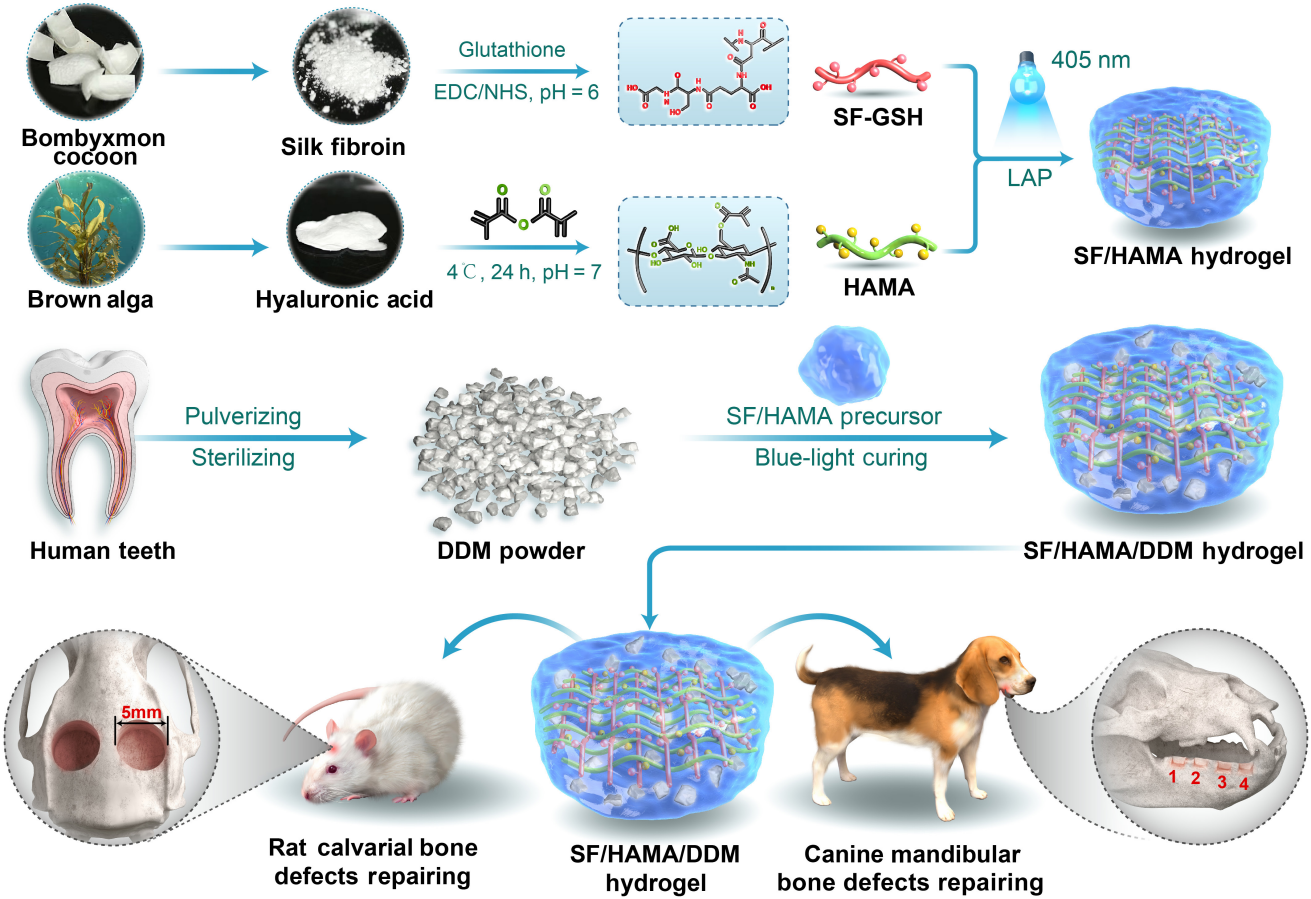
Preparation of composite bone substitutes and schematic diagram of repairing bone defects.

Therefore, a biomimetic, osteogenic, and plastic novel composite bone graft substitute (SF/HAMA/DDM hybrid hydrogel) was developed in this study using a rapid and clinically applicable strategy. The incorporation of the hydrogel endowed DDM particles with plasticity and could be rapidly gelatinized under blue light. Unlike conventional hydrogels lacking osteogenic cues, the SF/HAMA dual-network hydrogel combined with DDM particles endows the material with the ability to mimic the hierarchical structure of natural bone, demonstrating both osteoinductive and osteoconductive properties. To validate our hypothesis, we first optimized the ratio of SF/HAMA/DDM hybrid hydrogel and material properties, investigated the biocompatibility of the composite bone graft substitute through in vitro and in vivo experiments, and evaluated its ability to guide cell osteogenic differentiation. Furthermore, the SF/HAMA/DDM bone graft substitute was filled into critical-size cranial defects in Sprague-Dawley (SD) rats and mandibular bone defects in beagles, respectively, to evaluate the effects of bone defect regeneration. Finally, the osteogenic ability of the SF/HAMA/DDM bone graft substitute was systematically researched. Figure 1 shows the research process. This highly biocompatible, rapidly solidifying novel composite bone graft substitute is expected to be made with a simple and applicable strategy and meet the challenges of bone defect treatment.

## Materials and Methods

### Preparation of SF-GSH

First, mulberry cocoons were cut into pieces and boiled in a solution of 0.4% (w/v) Na_2_CO_3_ for 30 min to remove the silk glue protein. The SF was then washed repeatedly with deionized water, and the fiber was drained and dried at 60 °C in a drying oven overnight. To obtain the SF solution, the fiber was dissolved at 70 °C for 4 h in a ternary solvent (CaCl_2_/CH_3_CH_2_OH/H_2_O = 1:2:8 in mole ratio). Next, the solution was dialyzed against deionized water for 3 days using a dialysis membrane (molecular weight cutoff [MWCO] 3,500 Da, Solarbio).

To obtain SF-GSH, a series of modifications were performed on the SF solution. First, the completed dialysis solution was placed in MES buffer at pH 6, together with the dialysis membrane. After 24 h, 1-(3-dimethylaminopropyl)-3-ethylcarbodiimide hydrochloride (0.2 to 0.4 mol/l) and N-hydroxysuccinimide (0.4 to 0.6 mol/l) were added into the solution. After 15 min of reaction at ambient conditions, reduced glutathione was added and SF was modified by mercapturization through a condensation reaction. After these steps, the solution was dialyzed for another 2 days and lyophilized to yield a solid SF-GSH.

### Synthesis of HAMA

HA was dissolved in deionized water to prepare a 2 wt% solution. *N*,*N*-dimethylformamide (DMF) is added at a volume ratio of deionized water:DMF = 3:2. Then, place the mixed solution in a condensation circulator at 4 °C. After the solution is well mixed, 2 ml of methacrylic anhydride is slowly added dropwise to the HA solution, and allow the solution react for 24 h at magnetic stirring. At the same time, the pH is adjusted to 8 to 9 with 1 mol/l NaOH. Then, add 0.1 mol NaCl to the solution, and after it has completely dissolved, pour the solution into 2.5 to 3 times the volume of anhydrous ethanol. The flocculent precipitate in solution is removed for centrifugation, and the centrifuged solid is then re-dissolved in deionized water. Upon completion of reaction, the HAMA solution is transferred into a dialysis membrane (MWCO 7,000 Da, Solarbio) and dialyzed against deionized water at ambient temperature for 3 days. When the dialysis was complete, the HAMA solution is lyophilized to generate a white porous sponge.

### Preparation of DDM

DDM is made from extracted teeth. The preparation procedure of DDM is as described previously [33]. Briefly, to remove the periodontal ligament, enamel, decayed areas, and the restorations from the extracted tooth, a high-speed dental handpiece (T3 Racer Midwest; Dentsply Sirona) was used. Subsequently, the tooth was ground and crushed into particles using a Bonmaker device (Korea Dental Solution Co. Ltd, Korea) to pick out pulp or root canal filling material. Following the sequential filtration process, dental enamel, cementum, and oversized dentin components were excluded, thereby isolating the target DDM particles. Finally, the fragments are placed into a bone preparation device that utilizes vacuum ultrasonic high-pressure sterilization, and different solutions (Liquid A: 3% hydrochloric acid, Liquid B: 10% hydrogen peroxide, and Liquid C: 80% ethanol) are used according to the manufacturing method. After steps such as demineralization and hydrogen peroxide sterilization rinse, granular DDM is ultimately obtained.

### Preparation of the SF/HAMA/DDM bone graft substitute

SF-GSH and HAMA were dissolved in phosphate-buffered saline (PBS) at a mass ratio of 1:1, 1:2, and 1:3, to generate prepolymer solutions. As the reduced glutathione grafted on the thiolated modified SF is prone to oxidation to oxidized glutathione, we added the reducing agent tris(2-carboxyethyl) phosphine hydrochloride to open the disulfide bond before preparing the hydrogel. For each bone graft substitute group, the prepolymer solution concentration was fixed at 5 wt%. Different masses of DDM were added to the solution, producing a series of mixtures with varying hydrogel-to-DDM mass ratios: 100:0 (100% hydrogel), 85:15 (85% hydrogel), 65:35 (65% hydrogel), and 50:50 (50% hydrogel). For each group, the concentration of LAP was 0.1 wt%. Then, the mixture was exposed to 365 to 405 nm blue light (Zigu Lighting Factory, China) for 30 s.

### Characterization of SF/HAMA/DDM

#### NMR test

The ^1^H NMR analysis was performed using a Bruker AVANCE III 500-MHz spectrometer (Switzerland) to characterize pure SF, modified SF (SF-GSH), HA, and methacrylated hyaluronic acid (HAMA). For each sample (10 mg of SF, SF-GSH, HA, or HAMA), 1 ml of deuterated water (D_2_O) was used to dissolve the material in an NMR tube prior to ^1^H NMR spectral acquisition.

#### SEM characterization

For scanning electron microscopy (SEM) characterization, the hydrogel samples were subjected to freeze-drying and cryo-fractured in liquid nitrogen to expose the fracture surfaces and subsequently cleaned with a nitrogen stream to remove residual debris. Samples were coated with platinum 90s (25 °C, 15 kV). The SEM instrument was operated at an accelerating voltage of 10 kV to observe the surface and the size of hole.

#### Mechanical test

Mechanical properties of the hydrogels were tested by Electronic universal material testing machine Instron5969 (Instron, USA). Some cylindrical hydrogel samples (*D* = 10 mm, *H* = 10 mm, *n* = 3) were prepared and tested at a rate of 1 mm/min with a 200 N load until failure. The deformation rate was set to 80% of the original height. Stress–strain curves were drawn based on the results.

#### Swelling property

The SF/HAMA hydrogel SR test sample (*n* = 3) was prepared and placed in deionized water at 37 °C for different periods of time before being taken out. After gently drying the water on the surface with absorbent paper, the sample was weighed as *W*_1_, and then freeze-dried and weighed again as *W*_2_. The average value of the 3 groups was calculated as SR, SR = (*W*_1_ − *W*_2_)/*W*_2_.

#### In vitro enzymatic degradation

The samples were prepared for degradation in vitro. The initial weight (*W*_0_) of the samples was measured with an electronic balance, and the samples were immersed in 3.0 ml of hyaluronidase solution. The samples were placed in a 37 °C constant-temperature oscillating incubator (100 rpm), and taken out at 24 h, 48 h, 3 days, 5 days, 7 days, 2 weeks, and 3 weeks. The water on the surface of the material was quickly absorbed with filter paper, weighed by an electronic balance, and the retained mass (*W*_t_) of the sample was tested and the degradation rate of the material was calculated.

### Biocompatibility of SF/HAMA/DDM

#### Leaching solution preparation and cell culture

Firstly, Dulbecco’s modified Eagle medium (DMEM) (50 ml) or minimum essential medium (MEM) (50 ml) complete medium was prepared, which was configured as follows: 45 ml of DMEM or MEM + 0.5 ml of penicillin/streptomycin + 5 ml of fetal bovine serum. Then, equal amounts of SF/HAMA, SF/HAMA/DDM-15, SF/HAMA/DDM-35, and SF/HAMA/DDM-50 samples were immersed in DMEM or MEM complete medium for 24 h at a sample:medium ratio = 1 g:17 ml, and the leachate was collected.

L929 mouse fibroblasts cultured in complete DMEM (Gibco, America) and human periodontal ligament stem cells were provided by Zhejiang Provincial People’s Hospital and cultured in complete MEM (Gibco, America). After 24 h of incubation at 37 °C in a 5% CO_2_ incubator, the medium was removed and the cells were cultured with the leaching liquor mentioned above.

#### Biocompatibility of SF/HAMA/DDM in vitro

The following experiments are used to test the in vitro biocompatibility of the bone graft substitute: cell proliferation assay, cell live/dead staining assay, and cell scratch assay. First, L929 cells were implanted on 96-well plates at a density of 5,000 cells per well, and 100 μl of complete medium was added to each well. The experimental design comprised 5 groups (4 experimental groups and 1 negative control group), each consisting of 5 technical replicates. After 24 h of culture in a 37 °C, 5% CO_2_ incubator, the medium was removed and 100 μl of leachate was added, respectively. The negative control group was added with 100 μl of DMEM complete medium. After 1, 3, and 5 days of culture, the leach solution was removed and 100 μl of cell counting kit-8 (CCK-8) reagent (10% W/V DMEM) was added to each well, and after 2 h of culture in a sterile incubator at 37 °C and 5% CO_2_, the light absorption value of each well at 450 nm was measured by enzyme labeling.

Cell viability under varying concentrations of SF/HAMA/DDM leachates was assessed using the Calcein-AM/PI dual fluorescence staining method (Beyotime, C2015S), which differentially labels live (green fluorescence, Calcein-AM) and dead cells (red fluorescence, PI). L929 was cultured in DMEM cell medium containing different proportions of leach solution in 48-well plates at 37 °C and 5% CO_2_, and the sample cells were collected. The cells were collected and washed 3 times with PBS. The cells were stained with 300 μl of live/dead solution and incubated at 37 °C for 15 to 30 min away from light. Then, the cells were washed with PBS to remove the dye. The results were examined by fluorescence microscopy.

L929 were co-cultured with the leachate of each group for 24 h, then digested and centrifuged with 0.25% trypsin, and the supernatant was discarded and added to the complete medium, and the concentration of the cells was adjusted to 4 × 10^5^/ml. The cell suspension was transferred with a pipette gun to a 6-well cell culture plate, with 2 ml in each well. The cells were cultured in the incubator at 5% CO_2_ and 37 °C for 24 h, and then scratched with the tip of the gun with a 200-μl tip perpendicular to the cell surface from one end of the well to the other end, respectively. Scratching from one end of the wells to the other end, and taking the scratches after 0, 24, and 48 h, respectively, the degree of cell migration between the scratches of different treatment groups was observed under the microscope.

Quantitative real-time polymerase chain reaction was performed on total RNA isolated from BMSCs using Trizol reagent (Invitrogen, USA). For mRNA detection, use the PrimeScript RT kit (Yeasen, China) according to the manufacturer’s instructions. For miRNAs, the corresponding cDNAs were obtained using a kit (Sangon, China) and PCR was performed using a LightCycler 480 SYBR Green I Master. Relative gene expression was determined by the 2^−ΔΔCt^ method and expression levels are shown as a percentage relative to GAPDH mRNA levels. Primer sequences are shown in Table S1.

#### Biocompatibility of SF/HAMA/DDM in vivo

To evaluate the in vivo biocompatibility of SF/HAMA/DDM, we performed subcutaneous implantation experiments on the back of rats. Male SD rats (8 weeks old, weight range of 270.0 to 300.0 g) were selected. After the surgical area was prepared and sterilized with iodophor, a 1.0-cm incision was made on each side of the midline of the rat’s back, and the material was implanted into a subcutaneous pouch-like structure by blunt dissection and the wound was sutured. After 1 week of implantation, the rats were sacrificed to remove the subcutaneous tissue surrounding the hydrogel and the individual organs were removed. The tissues were fixed in 4% paraformaldehyde for 48 h, and then underwent stepwise dehydration, paraffin embedding, sectioning, and hematoxylin and eosin (H&E) staining.

#### Osteogenic capacity of SF/HAMA/DDM in vitro

Periodontal ligament stem cells (PDLSCs) were cultured in MEM medium for 24 h and then further cultured in osteogenic induction medium (10 × 10^−3^ mol/l beta-glycerophosphate, 0.1 × 10^−3^ mol/l dexamethasone, and 0.25 × 10^−3^ mol/l ascorbic acid), and the ability of SF/HAMA/DDM to guide the osteogenic differentiation of the cells was assessed by ALP staining and alizarin red S (ARS) staining, 4, 7, 14, and 21 days after induction of osteogenesis.

### In vivo evaluation of SF/HAMA/DDM for bone defect repair

#### Animal model

SD rats were approved by the Ethics Committee of Zhejiang Provincial People’s Hospital (Ethics Permission No. 20230527175018933362). Male SD rats (8 weeks old, weight range of 270.0 to 300.0 g) were selected to be anesthetized by intraperitoneal injection (dose 30 mg/kg) using sodium pentobarbital/saline solution (2%), hair around the head was removed, the cranium was completely exposed, and an electric drill was used to drill 2 round defects about 2 mm deep and 5 mm large in the cranium into the bone graft substitute. The wounds were then sutured in layers, and all rats received injections of antibiotics for 3 days.

Beagles were approved by the Animal Ethics Committee of Zhejiang University of Traditional Chinese Medicine (Ethics License No. IACUC-20231016-02). Six adult male beagles weighing about 15 to 20 kg, aged about 10 to 13 months, with intact dentition and healthy periodontium were selected, and 4 premolar teeth were extracted on the right side of the mandible of each beagle and then sutured and healed for 2 weeks. The dogs were sedated by intramuscular injection of xylazine hydrochloride (0.2 ml/kg) into the medial hind limb and anesthesia was induced by intravenous injection of 3% sodium pentobarbital (0.3 ml/kg). The mandibular cusps to the proximal middle of the first molar were incised along the upper 1 cm of the vestibular sulcus with a razor blade and the flap was turned. Four separate critical-size wall defects of 8.0 mm (proximal–distal), 5.0 mm (vertical), and 5.0 mm (buccolingual) were formed on each side by bone drilling. The pre-prepared and sterilized bone graft substitute was then filled into the defect areas according to the grouping. The wounds were then closed in layers and all beagles received injections of antibiotics for 3 days.

#### Micro-CT evaluation

A total of 16 rats (4 in each group) were operated on and cranial tissue samples were collected at 8 and 16 weeks after surgery. Six beagles (*n* = 3) were operated on and mandibular tissue samples were collected at 4 and 8 weeks after surgery. The rat skulls and beagle mandibles were fixed in 75% alcohol for 3 days. These skulls and mandibles were then scanned using a micro-computed tomography (CT) device for 3D reconstruction, bone tissue volume/total tissue volume (BV/TV%), trabecular thickness (Tb.Th), and trabecular separation (Tb.Sp).

#### Statistical analysis

Data were presented as mean ± standard deviation. All statistical analyses were performed using GraphPad Prism 8 software (La Jolla, CA). Multiple comparisons between groups were conducted by one-way analysis of variance (ANOVA), 2-way ANOVA, and Tukey’s post hoc test. Statistical significance was defined as follows: *P < 0.05, **P < 0.01, ***P < 0.001, and ****P < 0.0001; ns, no significant difference.

#### Histological assessment

The specimens after micro-CT scanning were subjected to histological staining. The integration of the new bone, the remaining bone graft substitute, and the bone graft substitute with the new bone was observed by H&E staining, Masson staining, and Goldner trichrome staining, respectively. To produce paraffin tissue samples after surgery, rat skull tissue samples were collected after 8 and 16 weeks, and beagle mandible samples were collected after 4 and 8 weeks, decalcified and embedded, and then 5-μm-thick sections were prepared for staining.

## Results

### SF/HA/DDM bone graft substitute synthesis and characterization

**Fig. 2.**
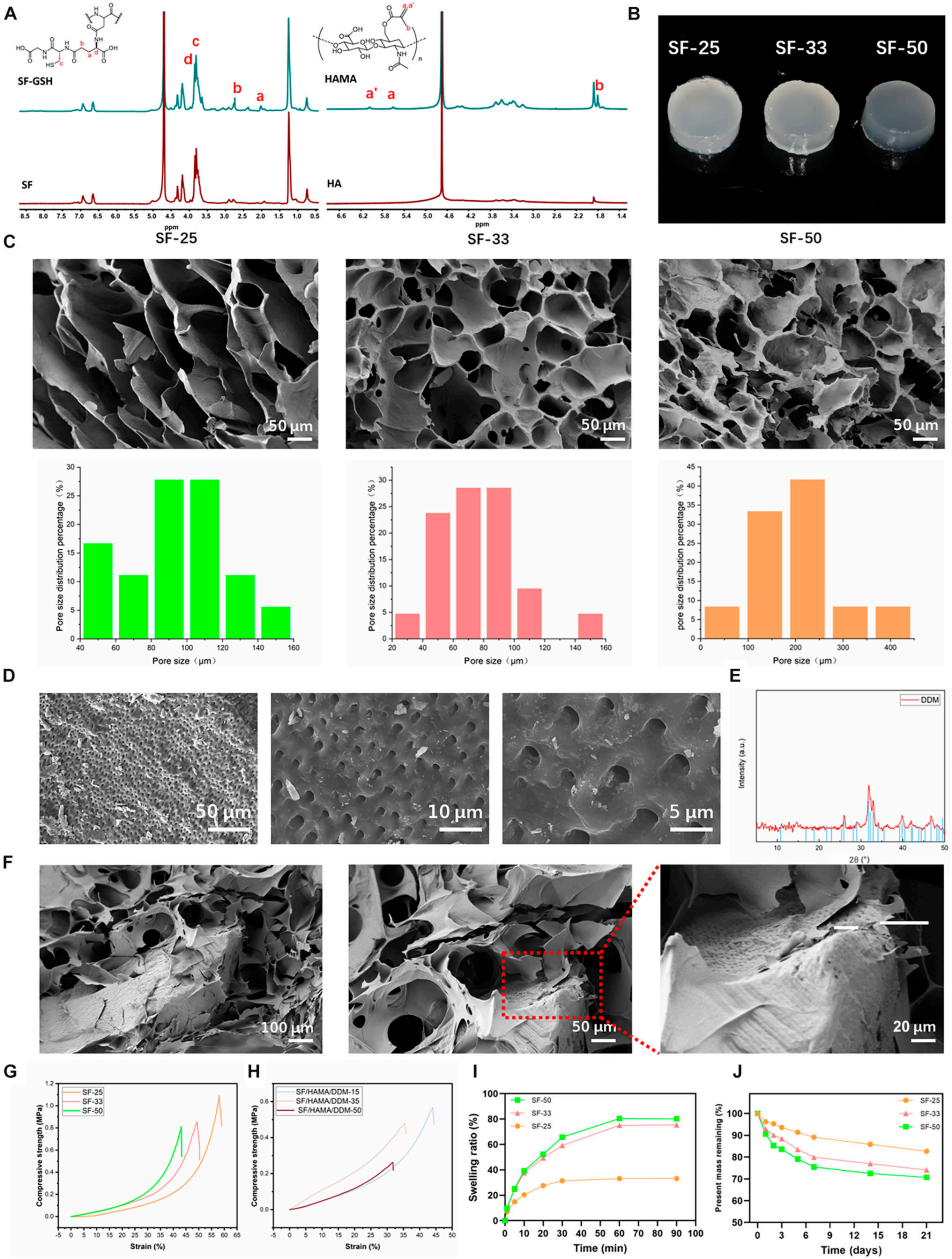
Characteristics of the SF/HAMA composite hydrogel. (A) ^1^H NMR spectra of SF-GSH and HAMA. (B) Hydrogel in different proportions. (C) Microporous morphology and pore size distribution of SF/HAMA hydrogel sections with different ratios. (D) Microstructure of DDM under different magnifications via electron microscopy. (E) DDM physical phase analysis. (F) The microporous morphology of the SF/HAMA/DDM section of the SF-25 group. (G) Mechanical properties of SF-25, SF-33, and SF-50 composite hydrogels. (H) Mechanical properties of SF/HAMA/DDM-15, SF/HAMA/DDM-35, and SF/HAMA/DDM-50. (I) Swelling properties of SF-25, SF-33, and SF-50 composite hydrogels. (J) Degradation properties of SF-25, SF-33, and SF-50 composite hydrogels. The scale bars in (C) are 50 μm; those in (D) are 50, 10, and 5 μm; and those in (F) are 100, 50, and 20 μm.

Figure 2A shows the ^1^H NMR spectrum of SF-GSH. The results show that compared with SF, SF-GSH has some characteristic peaks, which appear at 2.17, 2.56, 2.93, and 2.95 parts per million (ppm). These characteristic peaks indicate that GSH has successfully covalently bonded with SF. Among them, 2 hydrogen peaks are located at 2.93 and 2.95 ppm, corresponding to the methylene connected to the thiol group in GSH. We used the hydrogen peaks on the phenyl ring of SF (6.75 and 6.93 ppm) as reference peaks and calculated the degree of substitution of GSH on SF to be 17.99% through the analysis of the integral area of nuclear magnetic resonance.

Figure 2A shows the ^1^H NMR spectrum of HA modified by MA. In HAMA, weak diffraction peaks can be seen, which appear at 1.8, 5.6, and 6.1 ppm. These peaks correspond to the characteristic peaks of MA, respectively representing the methyl hydrogen and the 2 hydrogens on the double bond carbon of MA, indicating that MA has been successfully grafted onto the HA main chain. Using the methyl hydrogen on the -NHCOCH_3_ of the HA main chain as a reference peak, the degree of substitution of MA on HA was calculated to be 48% through the calculation of the integral area of nuclear magnetic resonance. The ^1^H NMR spectrum of HAMA is shown in Fig. 2A.

We prepared an SF/HAMA hydrogel through a thiol-ene click reaction for rapid cross-linking under blue light to validate the fast curing and formation of the hydrogel. By adjusting the mass ratios of HAMA to SF-GSH, we fabricated hydrogels with tunable cross-linking densities, designated as SF-25 (25% SF, 75% HAMA), SF-33 (33% SF, 67% HAMA), and SF-50 (50% SF, 50% HAMA), where the numerical suffix indicates the mass percentage of SF in the hybrid network (Fig. 2B). SEM and pore size distribution diagrams revealed a uniform, open porous structure in all hydrogels post freeze-drying. The cross-linking density of the SF hydrogel is inherently governed by the stoichiometric ratio between thiol groups (from the SF hydrogel) and methacrylate double bonds (from HAMA) via the thiol-ene click chemistry. Increasing the SF content to 50 wt% significantly enhanced the cross-linking density of the framework, resulting in smaller pore sizes, consistent with the pore size distribution analysis (Fig. 2C), which revealed that SF-25 exhibited a higher proportion of macropores (>100 μm) compared to SF-33 and SF-50. Given the need for higher porosity and larger pore size for improved osteogenesis, SF-25 was chosen for subsequent experiments.

Normal dentin tubules have a diameter of about 1 to 4 μm depending on their location [34], and when the DDM was observed under SEM (Fig. 2D), it was seen that the dentin tubules were more uniformly distributed with a pore size of about 2 μm. After demineralization, the main component of DDM is HAp [3]. The result of the Brunauer–Emmett–Teller test revealed that the specific surface area of DDM was 1.07 m^2^/g (Table S2). In addition, the results of element analysis are displayed in Fig. S1. Figure 2E shows a typical spectrum of DDM, where the characteristic peak containing HAp (JCDS09-0423) can be observed, and the narrowness of the diffraction peaks reflects a higher degree of crystallinity. SEM observation of SF-25 mixed with DDM showed that the hydrogel maintained its porous structure, with evenly arranged dentinal tubules and pores on DDM (Fig. 2F).

Mechanical property testing is a crucial component of performance evaluation in bone tissue engineering, as subpar mechanical properties can hinder the utilization of composite hydrogels within this field. In this study, the compression modulus of composite hydrogel and bone graft substitute was assessed, with the results depicted in Fig. 2G indicating that the mechanical strength of SF-25, SF-33, and SF-50 was measured at 0.38, 0.24, and 0.18 MPa, respectively. Furthermore, the addition of varying amounts of DDM resulted in a reduction in compression strength, with values of 90, 43, and 29 kPa observed for SF/HAMA/DDM-15, SF/HAMA/DDM-35, and SF/HAMA/DDM-50, respectively (Fig. 2H).

The swelling of hydrogel in PBS reached equilibrium within 30 min, with no further weight increase, which had a rate of about 30 wt% (Fig. 2I). The SF/HAMA hydrogel maintained its initial shape throughout the test, indicating its suitability as a bone graft substitute due to its physical and chemical stability. Furthermore, uncontrollable and quick degradation, due to the rapid and outburst breaking of cross-linked macromolecular chains, is a common issue for the steep decline of the mechanical property of natural macromolecular gels, affecting bone defect repair. Thus, adjusting the degradation time is key to the osteogenic effect of the composite hydrogel. Figure 2J shows the degradation rate changes of 3 composite hydrogels soaked in PBS for 0 to 21 days. The results indicate that the SF/HAMA hydrogel’s degradation rate is controllable and low (17.34% at 21 days), making it suitable as a bone graft substitute for bone tissue regeneration.

Owing to flexible SF/HAMA hydrogel, granular DDM particles could be efficiently fixed in the hydrogel matrix. The hybrid hydrogel can be feasibly processed on the anomalous bone defect and showed good adhesive on the tissue surface. The biofriendly blue light, which was broadly used in restorative dental surgeon, could rapidly cure the hydrogel, seal the opening wound, and prevent the loosing of DDM substitute. The detailed clinical operation record can be seen in the Supplementary Materials.

### SF/HA/DDM bone graft substitute biocompatibility detection

**Fig. 3.**
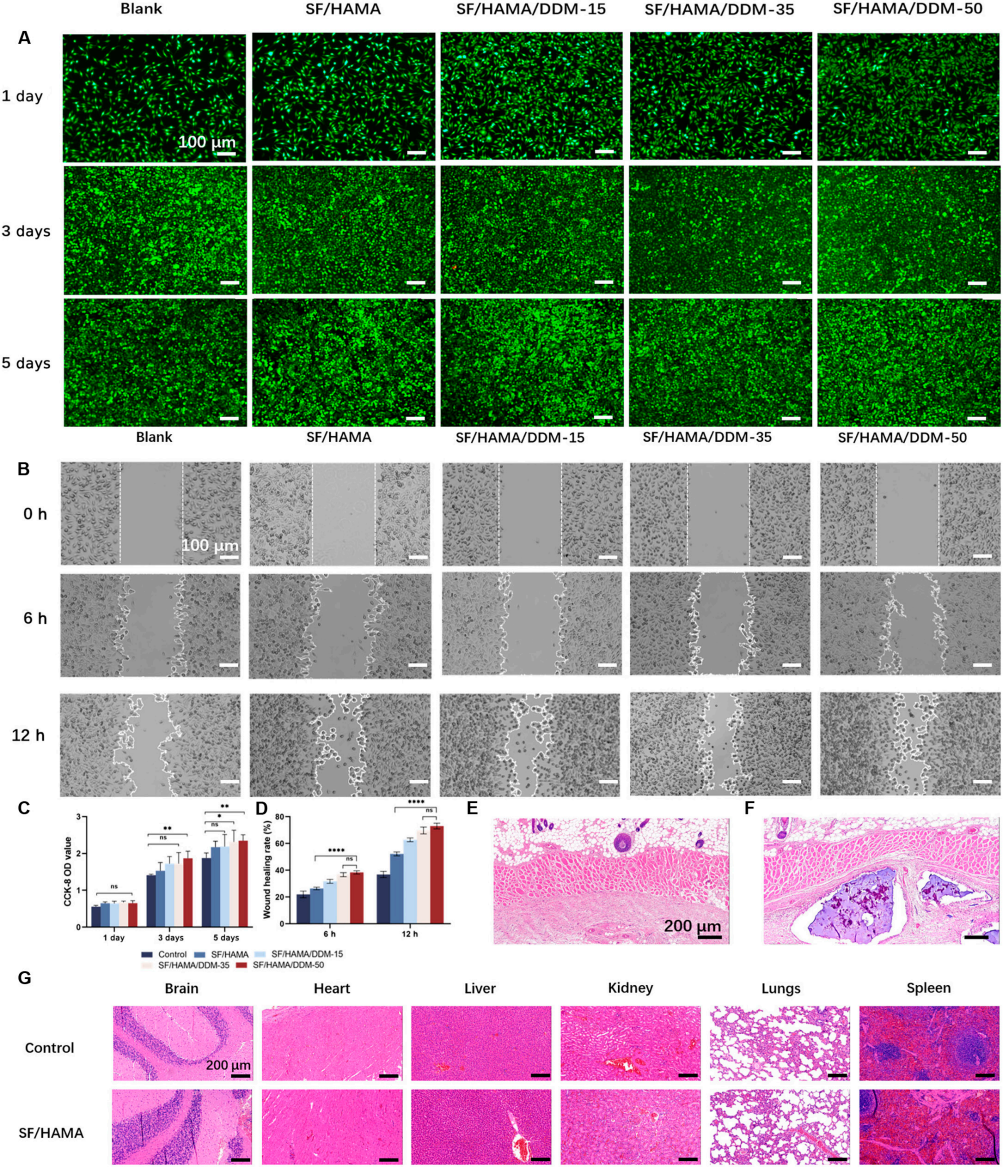
Biocompatibility of the SF/HAMA composite hydrogel. (A) Live staining and dead staining of SF/HAMA/DDM. (B) Scratch assay of PDLSCs in vitro. (C) Statistical plot of OD values for CCK-8 assay. (D) Quantitative analysis of the percentage of scratch area in scratch wound healing assay (*n* = 3). One-way analysis of variance (ANOVA) was used; NS, not statistically significant, *P < 0.05, **P < 0.01, ****P < 0.0001, *n* = 3. (E) H&E staining of subcutaneous tissue of normal SD rats. (F) Representative H&E-stained images of subcutaneous hydrogels after being implanted for 1 week. (G) In vivo experimental visceral toxicity analysis. The scale bars in (A) and (B) are 100 μm; and those in (E), (F), and (G) are 200 μm.

The SF/HAMA/DDM composites were prepared by blending SF-25 hydrogel (25% SF-GSH, 75% HAMA by mass) with DDM particles at mass ratios of 15%, 35%, and 50% relative to the total composite weight, denoted as SF/HAMA/DDM-15, -35, and -50, respectively (Fig. S2). The experimental groups consisted of a control group, SF/HAMA hydrogel, SF/HAMA/DDM-15, SF/HAMA/DDM-35, and SF/HAMA/DDM-50, and cell experiments all used leachate. Live/dead staining confirmed the activity of periodontal ligament stem cells (Fig. 3A). Although a small percentage of cells died in all 5 groups, the majority of cells divided at a normal rate and with acceptable morphology, indicating good cell compatibility. A scratch test was performed to examine the effect of the composite bone graft substitute on cell migration, a key factor in bone regeneration (Fig. 3B). The SF/HAMA/DDM-35 and SF/HAMA/DDM-50 groups showed stronger migration activity, promoting scratch healing. The statistical results presented in Fig. 3D indicate that SF/HAMA can promote cell migration to a certain extent. Furthermore, the addition of DDM had a synergistic effect, further promoting scratch healing. The CCK-8 test also demonstrated the low cytotoxicity of the hydrogel (Fig. 3C). After 1 day, all hydrogel groups supported comparable cell proliferation, and over time, a progressive increase in cell growth was observed in all groups. The cell viability level of the hydrogel group containing DDM was markedly higher than that of the control group at different time intervals. The highest number of cells was found in SF/HAMA/DDM-50 among all groups.

The hydrogel was implanted subcutaneously in the backs of the rats, and 1 week after implantation, the hydrogel degraded slightly and was encapsulated by fibrous connective tissue. Most of the hydrogel remained at the implantation site. The group with the implanted hydrogel did not exhibit obvious inflammatory cell infiltration or acute inflammatory reaction when compared to normal subcutaneous tissue (Fig. 3E and F).

### Evaluation of SF/HAMA/DDM composite bone graft substitute’s ability to promote osteogenesis

Periodontal ligament stem cells, as mesenchymal stem cells, have multidirectional differentiation potential [35], and the biological properties of bone graft substitute correlate with their ability to participate in osteogenic differentiation. The key to assessing the performance of bone graft biomaterials is to determine whether these materials can stimulate osteogenic differentiation of periodontal ligament stem cells with osteoblasts. Therefore, the experiments in this section aim to confirm and characterize the osteogenic potential of this bone graft substitute. The main subgroups were blank, mineralized, SF/HAMA, SF/HAMA/DDM-15, SF/HAMA/DDM-35, and SF/HAMA/DDM-50 groups.

**Fig. 4.**
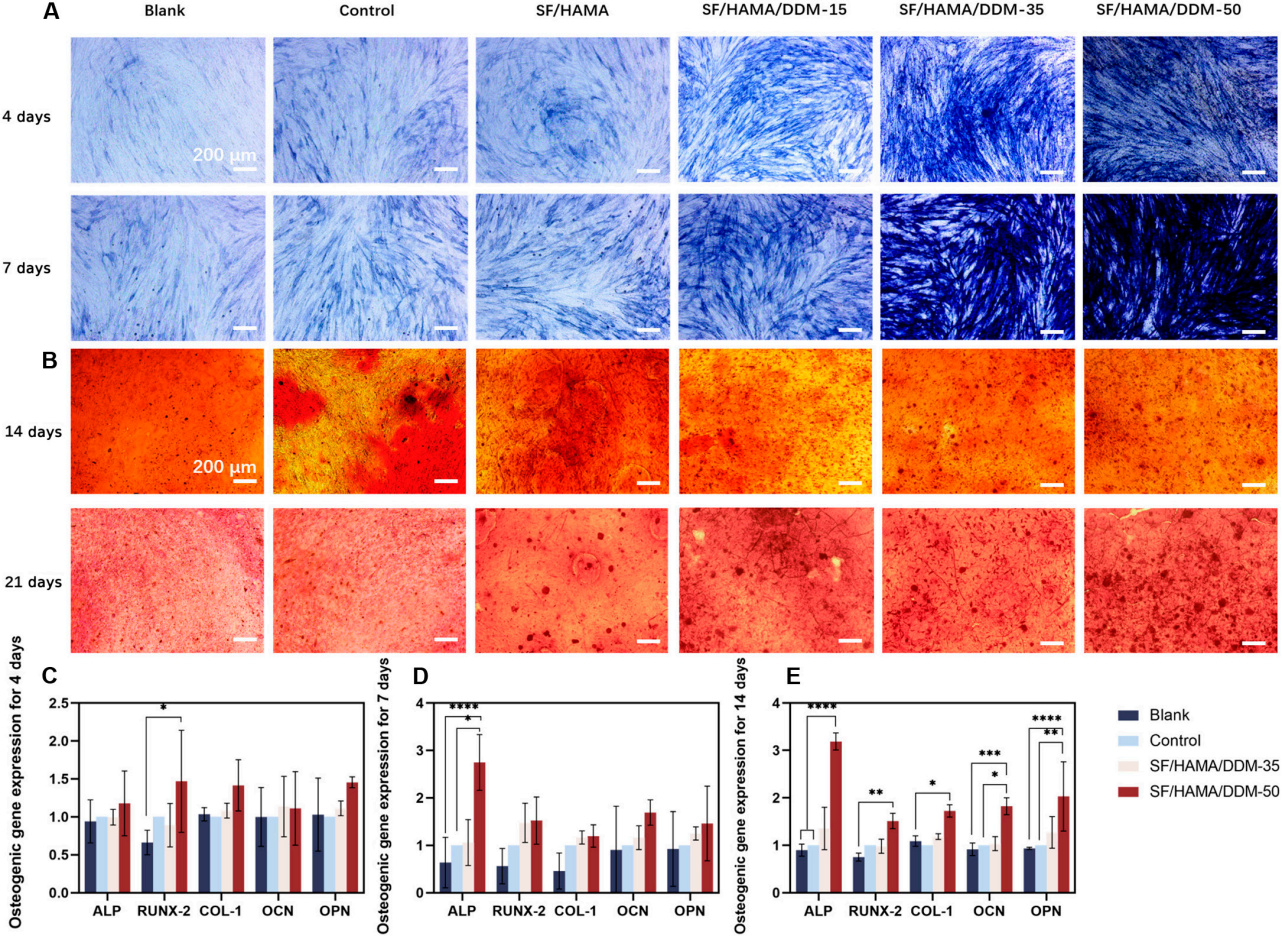
The pro-mineralization capacity of the SF/HAMA composite hydrogel. (A) ALP staining and (B) Alizarin red S staining of PDLSCs. Relative osteogenic gene expression of cells after 4 days (C), 7 days (D), and 14 days (E) of culture on the composite bone graft substitute (using 2-way ANOVA; NS, no significant difference, *P < 0.05; **P < 0.01; ***P < 0.001; ****P < 0.0001, *n* = 3). Scale bars in (A) and (B) are 200 μm.

First, periodontal ligament stem cells inoculated on this bone graft substitute for 4 and 7 days, and then stained with ALP under the induction of osteogenic conditioned medium. Figure 4A shows the microscopic images of ALP staining of periodontal ligament stem cells after 4 and 7 days of culture in hydrogel leachate. Compared with the blank group and the SF/HAMA group, the SF/HAMA/DDM group had a larger staining area, the staining intensity increased, the positive area widened with increasing DDM content, and the SF/HAMA/DDM-50 group presented the most pronounced increase. We also performed experiments using mouse embryo osteoblast precursor cells (MC3T3-E1) and obtained similar results (Fig. S3).

Secondly, ARS was used to stain periodontal ligament stem cells inoculated on the bone graft substitute on the day 21. ARS staining on day 21 demonstrated that the SF/HAMA/DDM-50 and SF/HAMA/DDM-35 groups exhibited greater calcium deposition (indicated by staining intensity) compared to the control and low-DDM groups, with SF/HAMA/DDM-50 showing the highest mineralization efficiency. These results indicate that the SF/HAMA/DDM composite bone graft substitute is a promising material for bone tissue regeneration.

Figure 4D and E illustrate the gene expression of composites during the osteogenesis process. Runx-2 and ALP are 2 typical markers often utilized for early osteoblast differentiation [36,37]. Runx-2 expression is directly correlated with both osteoblast activity and osteogenic differentiation [36]. The figure shows the evaluation of Runx-2 expression levels by the composites on days 4, 7, and 14, respectively. Within each time point, SF/HAMA/DDM composites up-regulated Runx-2 expression relative to the control group. Furthermore, the early differentiation phase of the skeleton is shown by the rise of the early marker ALP from day 4 to day 7.

Col-1 is a protein in the extracellular matrix that promotes osteoblast adhesion and development and contributes to bone maintenance and repair [38]. On day 14, Col-1 levels were found to be markedly higher in both SF/HAMA/DDM groups than in the control group. In addition, it has been shown that osteocalcin (OCN) is a marker for the stage of bone mineralization and can be expressed in mature osteoblasts [36]. OCN expression was markedly increased on day 14. The results were consistent with the up-regulation of OCN levels and enhanced mineralization of bone tissue. Expression of OPN in the experimental group was not statistically significant relative to the control group at 4 and 7 days and was statistically different at 14 days. Our preliminary experiment results suggest that bone graft substitute may increase the proliferation and osteogenic differentiation of periodontal ligament stem cells compared to other groups. This is supported by the expression of important osteogenic genes.

### Evaluation of the SF/HAMA/DDM composite bone graft substitute’s ability to treat large bone defects in vivo

**Fig. 5.**
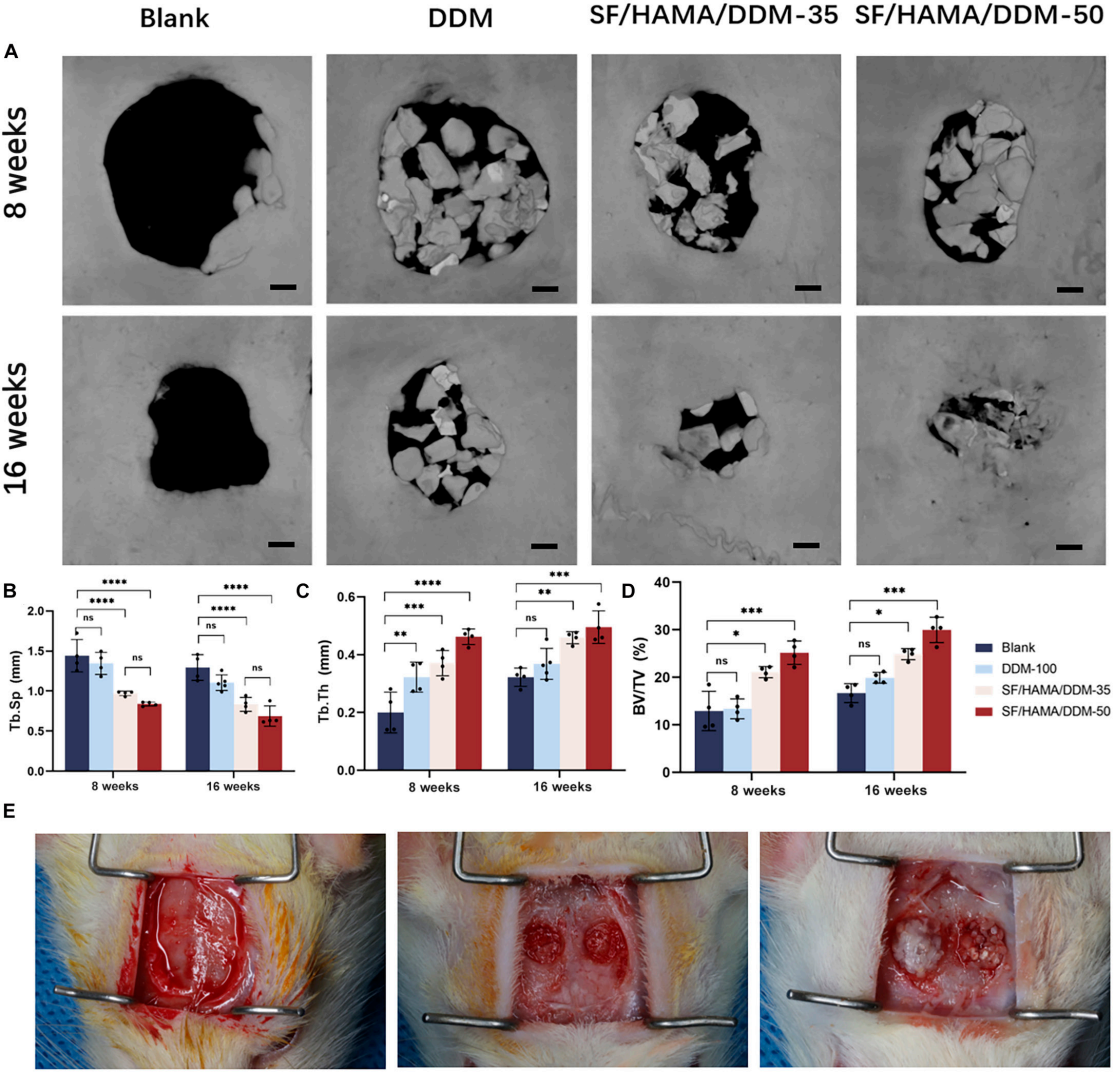
Experimental repair of cranial defects in rats. (A) 3D reconstruction of rat calvarial defect at 8 and 16 weeks. (B) Quantification of trabecular thickness; trabecular separation (Tb.Sp), (C) bone surface; trabecular thickness (Tb.Th), and (D) bone volume/total volume (BV/TV) from the micro-CT results (*n* = 4) (2-way ANOVA; NS, no significant difference; *P < 0.05; **P < 0.01; ***P < 0.001; ****P < 0.0001, *n* = 3). (E) Surgical photographs of the repair of cranial defects in rats using composite bone graft materials. The scale bars in (A) are 500 μm.

The in vivo performance of the SF/HAMA/DDM composite bone graft substitute was evaluated through a series of experiments. The bone graft substitute was implanted into rat skull defects for 8 and 16 weeks, and the rats were euthanized under excessive pentobarbital anesthesia and sampled for micro-CT scanning. The 3D reconstruction results demonstrate the cranial bone healing outcomes in rats at 8 and 16 weeks. (Fig. 5A). The blank group and DDM group showed no obvious trend of defect reduction, with only a small amount of new bone forming at the edge of the bone defect. The SF/HAMA/DDM-35 and SF/HAMA/DDM-50 groups showed better osteogenesis effects at each time point, markedly reducing the area of rat skull defects. After 16 weeks, the SF/HAMA/DDM-50 group was completely ossified, with nascent bone tissue almost completely filling the defect area. Quantitative data (Fig. 5B to D) showed that compared with the blank group and DDM group, the SF/HAMA/DDM-35 and SF/HAMA/DDM-50 groups had significant statistical differences in indicators such as BV/TV% and Tb.Th. For Tb.Sp, the SF/HAMA/DDM-35 and SF/HAMA/DDM-50 groups had no statistical difference, but were markedly lower than the control group and DDM group. Compared with the SF/HAMA/DDM-35 group, more new bone formation at the edge of the defect could be observed in the samples of SF/HAMA/DDM-50 group, and new bone formation could also be seen in the central area of the defect.

**Fig. 6.**
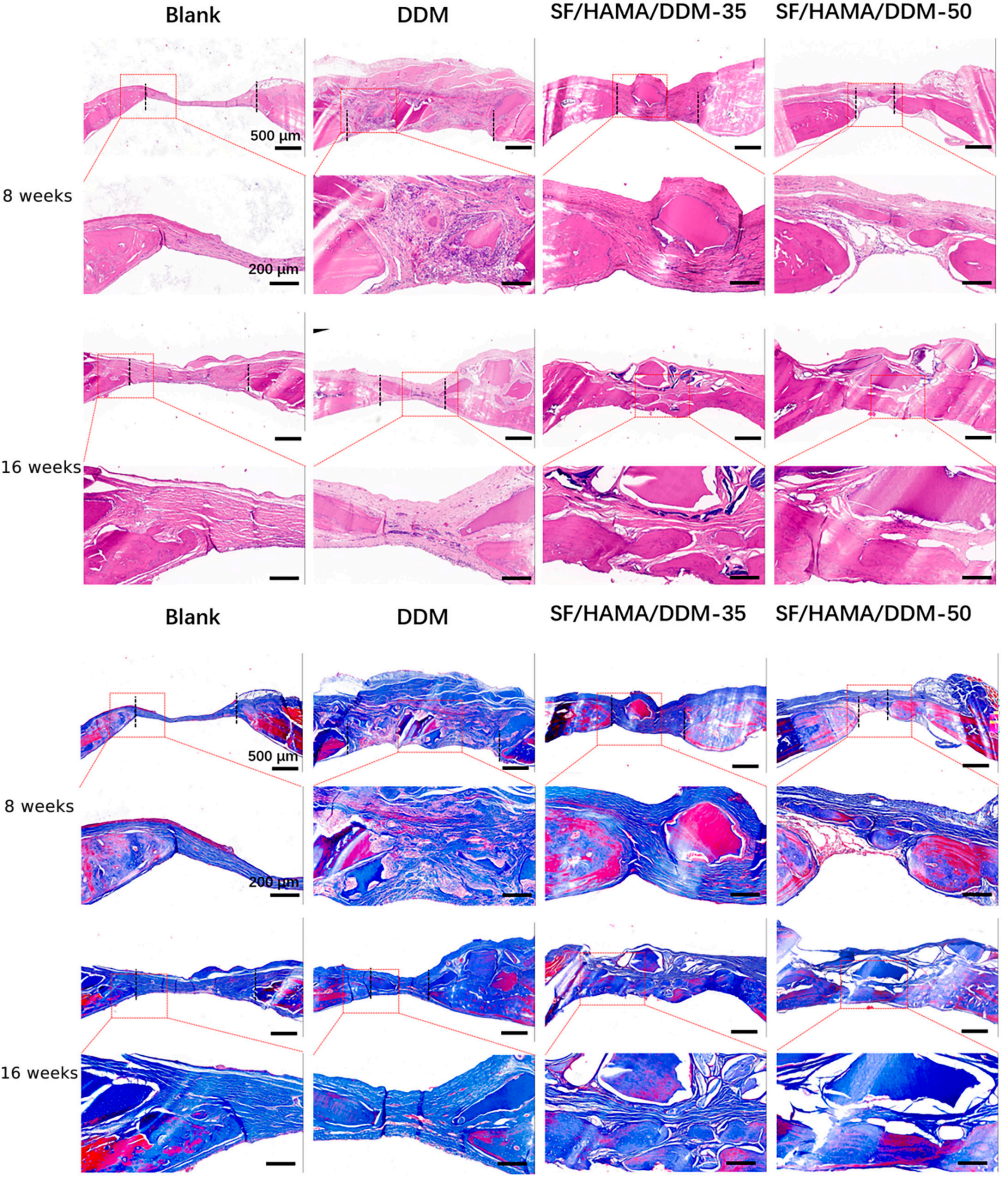
Histologic examination of the repair of cranial defects in rats. Enlarged images of H&E staining and Masson staining of rat skulls at 8 and 16 weeks postoperatively. The scale bars in the odd-numbered rows are 500 μm, and those in the even-numbered rows are 200 μm.

The osteogenic potential of the SF/HAMA/DDM composite was assessed histologically (H&E and Masson staining) at 8 and 16 weeks after implantation in rat cranial bone defects (Fig. S4). The results of H&E and Masson staining 8 weeks after surgery (Fig. 6) showed that the bone defect sites of all groups were filled with a layer of fibrous connective tissue, which contained fibroblasts and blood vessels. In the blank group, no obvious new bone formation was observed at the end of the bone defect. In the DDM group, DDM particles were surrounded by fibrous tissue, and only a small amount of new bone was seen forming at the edge of the defect. Compared with the blank group and negative control group, the SF/HAMA/DDM-50 and SF/HAMA/DDM-35 groups had more new bone formation, markedly shortening the distance of bone defects on both sides. After 16 weeks of surgery, the blank group still had less new bone at the defect site, and a large amount of fibrous connective tissue was still filled in the defect. The DDM group had more new bones than 8 weeks after surgery, immature woven bone and mature trabeculae were mixed, DDM particles were partially absorbed, but more fibrous connective tissue could still be seen in the defect area. The SF/HAMA/DDM-35 and SF/HAMA/DDM-50 groups were basically completely healed at 16 weeks, the defect area was replaced by bone tissue (which was basically mature trabecular bone), blood vessels could be seen in the bone tissue, and fibrous connective tissue was covered on the bone tissue.

**Fig. 7.**
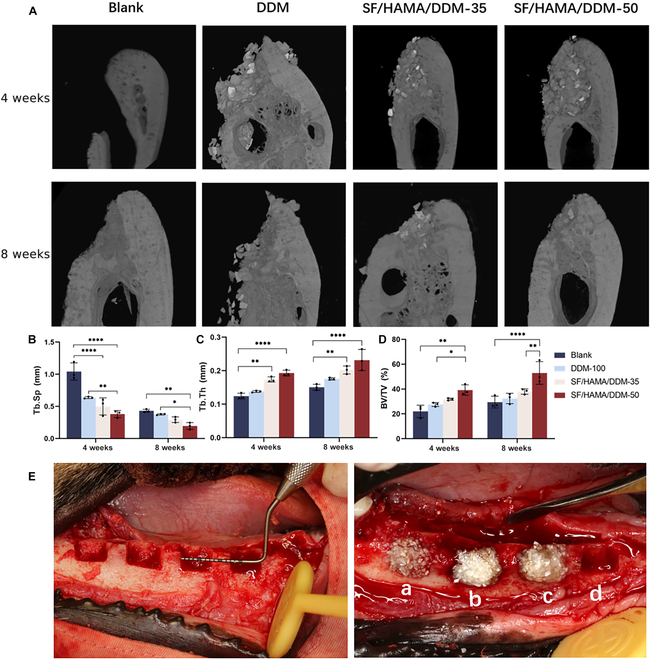
Experimental bone repair of mandibular defects in beagles. (A) Micro-CT 3D reconstruction of mandibular defects in beagles at 4 and 8 weeks. Quantification of (B) trabecular separation (Tb.Sp), (C) trabecular thickness (Tb.Th), and (D) bone volume/total volume (BV/TV) from micro-CT results (*n* = 3) (2-way ANOVA; NS, no significant difference; *P < 0.05; **P < 0.01; ****P < 0.0001, *n* = 3). (E) Surgical procedure for implantation of bone graft material into mandibular defects in beagles. (a) DDM; (b) SF/HAMA/DDM-50; (c) SF/HAMA/DDM-35; (d) Blank.

The bone graft substitute was also implanted into beagles’ mandibular defects for 4 and 8 weeks, and the dogs were euthanized under excessive pentobarbital anesthesia and sampled for micro-CT scanning (Fig. 7). The 3D reconstruction results of micro-CT at 4 weeks showed that new bone tissue could be seen at the bone defect site of all groups, but the blank group was still vacant, with obvious defects, and the rest of the groups could see DDM filling in the defect site, and the boundary of the defect site was relatively vague. After 8 weeks, the blank group’s defect was smaller than before, but it was still relatively vacant. The DDM part of the SF/HAMA/DDM-35 and SF/HAMA/DDM-50 groups was absorbed, and the osteogenesis was more obvious, and the new bone filled the defect area. Quantitative analysis data showed that as time increased, the BV/TV% and Tb.Th of all groups increased. At each time point, the BV/TV% and Tb.Th of the SF/HAMA/DDM-35 and SF/HAMA/DDM-50 groups were significantly higher than the blank group and the DDM group. For Tb.Sp, the SF/HAMA/DDM-50 group had the lowest Tb.Sp, indicating that the new bone in this group has the highest bone density.

**Fig. 8.**
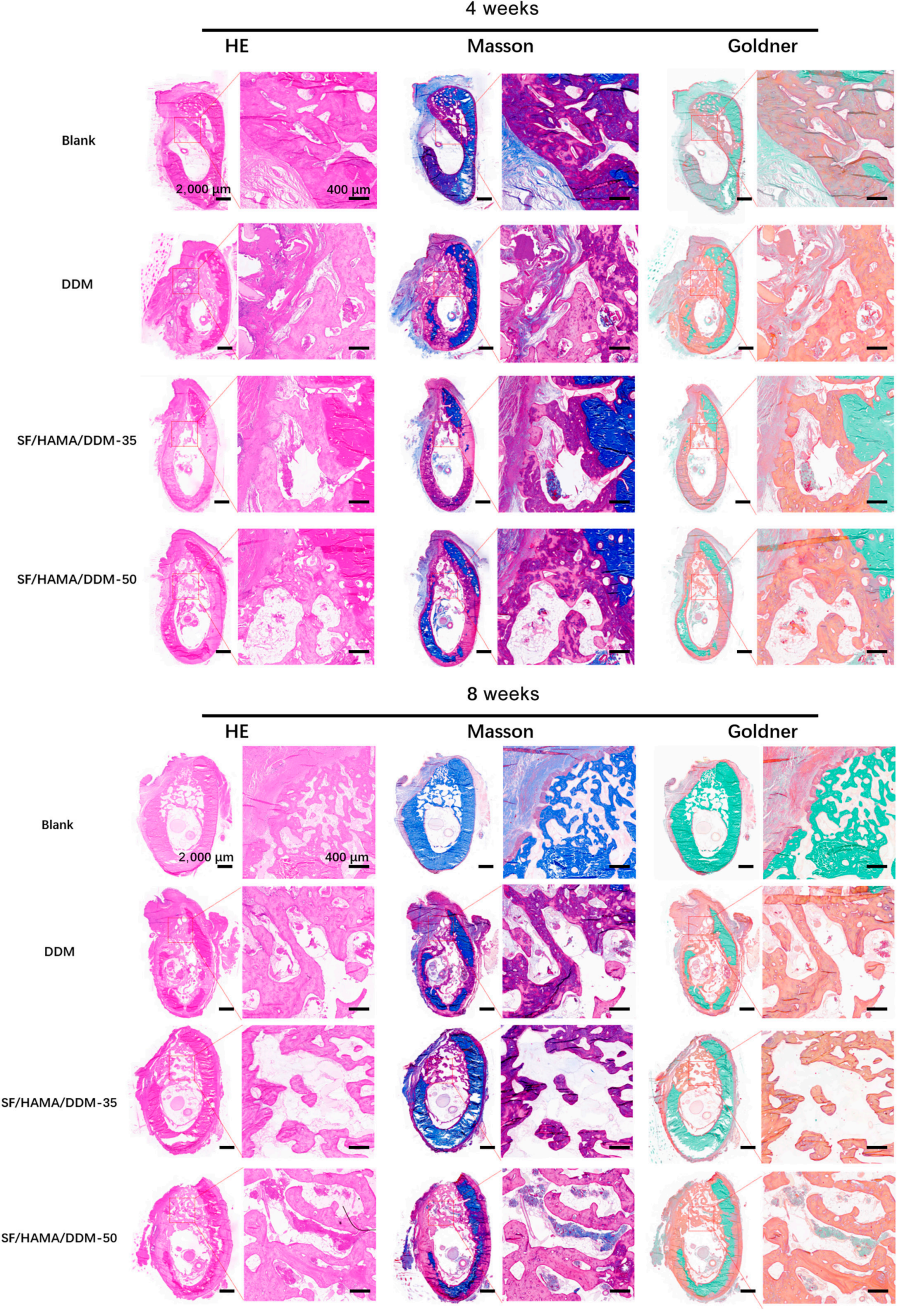
Histologic testing of bone repair for mandibular defects in beagles. Enlarged views of H&E, Masson, and Goldner stains of mandibular defects in beagles at 4 and 8 weeks postoperatively. The scale bars in odd-numbered columns are 2,000 μm and those in the even-numbered columns are 400 μm.

Through H&E, Masson, and Goldner trichrome staining (Fig. 8), the ability of the SF/HAMA/DDM composite bone graft substitute to repair beagle dog mandibular defects was evaluated. H&E staining revealed the basic histoarchitectural features of regenerated tissues. Nuclei were stained deep blue/purple by hematoxylin, while cytoplasmic components and extracellular matrices (e.g., collagen) exhibited pink/red eosinophilic coloration. Following Masson’s trichrome staining, muscle fibers are stained red, while collagen fibers appear green/blue. Goldner’s trichrome specifically highlighted bone mineralization dynamics: mineralized bone matrix appeared green/blue, while unmineralized osteoid (newly formed bone) was stained red.

After 4 and 8 weeks, new bone formation was found at the defect site of all samples. Macroscopically, in the control group, woven bone was observed at 4 weeks, and more cortical lamellar bone was observed at 8 weeks, and the mandibular volume was obviously insufficient. The SF/HAMA/DDM-35 and SF/HAMA/DDM-50 groups mainly had woven bone at 4 weeks, and at 8 weeks, the 2 groups showed more mature bone between the particles in the regeneration area.

In our study, the biosafety and in vivo results of this bone graft substitute verified good bone regeneration ability. Overall results showed that bone regeneration could be induced more effectively when DDM was doped into the hydrogel. Therefore, the biomimetic, biocompatible, and bioactive SF/HAMA/DDM bone graft substitute is an alternative solution for the treatment of severe bone defects.

## Discussion

Bone grafts are widely used to promote the repair and regeneration of bone defects. Their efficiency depends on the bioactivity of the bone graft substitute and its ability to promote osteogenesis [39]. Bone tissue engineering hydrogels have the potential to serve as an ideal solution for treating bone healing and bone defects. They act as a platform that integrates bone graft substitute and osteoinductive components, effectively promoting bone formation and healing by leveraging the advantages of the materials. In our study, based on a tissue engineering strategy, we developed an SF/HAMA dual-network hydrogel and introduced it into DDM to construct an SF/HAMA/DDM composite bone graft substitute. This combination exploits the plasticity of the hydrogel and the osteoconductive and osteoinductive properties of DDM. The bone graft substitute exhibited good osteoinductive capacity both in vitro and in vivo. Similarly, Bao et al. [19] demonstrated that the incorporation of optimal ratio of fibrin glue with DDM improves its osteogenic activity. The observed synergistic effects align with previous research by Sultan and Jayash [20] and Munir et al. [40], which documented the collaborative efficacy of biomaterial interactions in regenerative processes.

In this study, the first problem to solve is developing a hydrogel with good hydrophilicity and biocompatibility that also provides DDM with plasticity and high mechanical strength. Poor mechanical properties are a major disadvantage of natural polymers, making them unsuitable as load-bearing bone graft substitutes for large bone defects [41], which will also limit the prospect of their clinical application prospects. Recently, Bao et al. [19] evaluated the biological effects and osteoinductivity of the DDM-fibrin glue at an optimal ratio on bone healing from a rabbit calvarial defect model. However, its mechanical properties were not evaluated. Moreover, the findings would be necessary to validate in medium- or large-animal models. To address this issue, we chose SF and HA to construct a dual-network hydrogel. SF is commonly used in bone tissue engineering due to its exceptional biocompatibility and remarkable strength. As a natural macromolecule, it has garnered the attention of researchers because of its favorable biocompatibility and biodegradability. In this study, SF and HA were modified by mercapturization and double bonding processes using amide condensation and esterification reactions, respectively. The aim was to achieve a dual-network hydrogel of SF-GSH and HAMA. The resulting hydrogels exhibit excellent mechanical properties due to the cross-linking of 2 different networks. One network is more cross-linked and stiffer, while the second network is less cross-linked but more ductile. When faced with external pressure, the rigid network is the first to break, efficiently utilizing some of the energy.

We first used amide condensation and esterification reactions for mercapto and double bond modifications of sericin protein and HA, respectively, to obtain mercapto modified sericin protein (SF-GSH) as well as HAMA. The two were mixed to make the precursor liquid, and the composite osteogenic bone graft substitute with plasticity was obtained by mixing the precursor liquid with DDM. Under the conditions of initiator and blue light, the hydrogel wrapped with DDM was rapidly photocured by the mercapto-alkene clicking reaction, and the morphologically stable SF/HAMA/DDM composite bone graft substitute was obtained in only 30 s. The composite bone graft substitute with SF/HAMA was also modified by the mercapto-alkene clicking reaction under the conditions of blue light. The gelling properties of hydrogels have been verified in our previous study [42]. The compressive properties of the composites were partially reduced after the addition of DDM compared with pure hydrogel and were further reduced with higher DDM content. This may be due to the fact that the DDM particles with a diameter of 425 to 800 nm were selected in this experiment, and the DDM is harder than hydrogel, with irregular edges, which cut the hydrogel to a certain extent when compressed. However, overall, the compression performance of the composites is still above 20 kPa. The stiffness of the matrix material has been shown to be one of the key determinants in promoting stem cell differentiation [43], with stem cells tending to differentiate into lipids on softer matrices [44], whereas materials with greater stiffness (11 to 30 kPa) can induce the assembly of F-actin in stem cells as well as the contraction of the actinoglobulin cytoskeleton. This sequence of processes triggers the nuclear translocation of the Yes-associated protein (YAP)/transcriptional coactivator with PDZ-binding motif (TAZ) pathway, which promotes the osteogenic differentiation of stem cells [45,46].

Consistent with expectations, the SF/HAMA/DDM composite bone graft substitute showed good bone regeneration capacity both in vivo and ex vivo. The osteogenic potential of the composite bone graft substitute was assessed by ALP staining of periodontal ligament stem cells at 4 and 7 days, with ALP expression serving as a hallmark of early osteogenesis [46,47], and the results showed a positive correlation between the amount of DDM and the mineralization effect; i.e., the higher the amount of DDM, the darker the color of ALP staining. Mineralization of periodontal ligament stem cells at 14 and 21 days was subsequently analyzed using ARS staining. Consistent with previous results, the SF/HAMA/DDM-50 group with the highest DDM content promoted the formation of the most calcium nodules under the same culture conditions. Possible reasons for this result are the osteogenic differentiation-promoting role of DDM [2] and the fact that dentin tubules on the surface of DDM have been shown to be effective delivery channels for growth factors [14]. During demineralization, DDM retains a large number of naturally occurring BMPs, in particular BMP-2 [2,19], a potent osteoinductive factor included in the TGF-β superfamily, which stimulates the differentiation of stem cells to osteoblasts [48]. As a key regulator of early osteogenic differentiation, Runx-2 exhibits dynamic expression levels and function over time. Its expression is intricately linked with other osteogenic markers, such as ALP and COL-1, in a complex interplay. Despite the apparent increase in Runx-2 expression in the SF/HAMA/DDM-50 group compared to the blank group on day 7, the difference did not reach statistical significance. The observed transient decrease on day 7 may be indicative of a transitional phase, where Runx-2 expression temporarily down-regulated as the cells transition to a different stage of differentiation. This observation may also be partly attributable to inherent variations in cellular states, as well as inconsistencies arising from batch-to-batch preparation of the mineralization induction medium. In addition, BMP-2 increases ALP activity and induces bone regeneration in areas of effective oral and maxillofacial bone defects by enhancing the expression of osteogenic genes [49]. Therefore, the high content of DDM may have provided more BMP-2 and thus stimulated bone regeneration more effectively.

To verify the ability of SF/HAMA/DDM composite bone graft substitute to repair large bone defects in vivo, SF/HAMA/DDM-35 and SF/HAMA/DDM-50 were selected due to their superior in vitro osteogenic effect. The composite bone graft substitute was then filled into the critical bone defect area of rat skull and shaped with in situ light curing. The micro-CT results indicate that the bone regeneration volume and Tb.Th were higher in the SF/HAMA/DDM group and the DDM group, compared to the blank group and the DDM group, after 8 and 16 weeks of transplantation. The results of the micro-CT analysis indicate that the SF/HAMA/DDM-filled rats had higher bone regeneration volume and Tb.Th and lower Tb.Sp after 8 and 16 weeks of transplantation, compared to the blank and DDM groups. The SF/HAMA/DDM-50 group demonstrated the best ability to repair the defects. Histological analysis further confirmed the excellent bone tissue regeneration ability of the SF/HAMA/DDM composite bone graft substitute. Large bone defects were prepared in the right jawbone of beagle dogs to verify the osteogenic capacity of this composite in large animals. The bone graft substitute’s plasticity and in situ cross-linking properties offer new possibilities for irregularly shaped bone defects encountered during surgery. The bone graft substitute fills the bone defect area and maintains its shape, preserving local bone tissue without the need to trim irregular edges. The results of the 8-week micro-CT with histological sections were consistent with previous findings in the rat model. The SF/HAMA/DDM-35 and SF/HAMA/DDM-50 composite bone graft substitute induced more new bone generation, indicating that the SF/HAMA/DDM composite bone graft substitute can stimulate bone regeneration both quantitatively and qualitatively. The improved composite bone graft substitute may have better repair capabilities than DDM alone due to the hydrogel encapsulating granular bone powder, which is shape-fixed after curing. In contrast, DDM alone tends to escape [18]. In vivo experiments support the hypothesis that the SF/HAMA/DDM composite bone graft substitute can effectively promote bone regeneration as bionic and osteogenic tissue engineering materials.

In conclusion, our study developed a multifunctional hydrogel composite that can be used as an effective alternative for large-scale bone tissue regeneration. The results of this study demonstrate the promising application of SF/HAMA/DDM bone graft substitutes in repairing bone defects at both cellular and animal levels.

## Data Availability

All other relevant data supporting the main findings of this study can be found in the article and its Supplementary Materials or obtained from the corresponding authors upon reasonable request. The source data are provided with this article.
