# Supplementary material for: Prominent Alveolar Bone Graft Substitute Derived from Silk Fibroin/Hyaluronic Acid/Demineralized Dentin Matrix Hybrid Hydrogel
Source: Biomater Res. 2025 Aug 19;29:0243. doi: 10.34133/bmr.0243 (PMC12364377; doi:10.34133/bmr.0243)
Supplement: Supplementary 1 — Figs. S1 to S4 Tables S1 and S2 [file bmr.0243.f1.docx]

Supplementary Materials for

**Prominent alveolar bone graft substitute derived from silk fibroin/hyaluronic acid/demineralized dentin matrix hybrid hydrogel**

Runzhi Chen *et al.*

*Fan Yang. Email: yangfan@hmc.edu.cn

**This PDF file includes:**

Figs. S1 to S4

Table S1 to S2


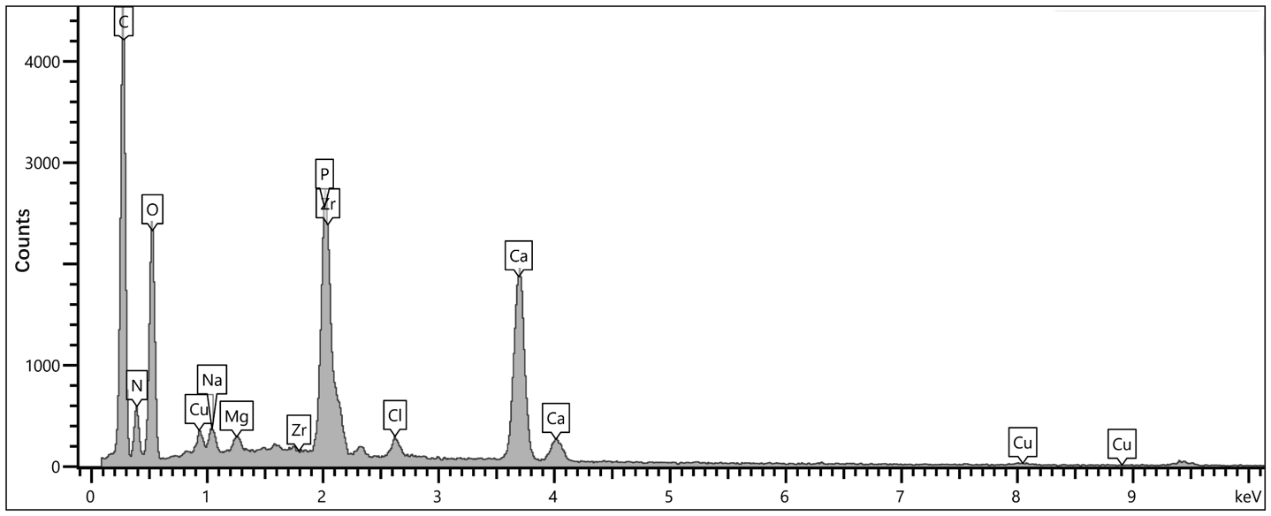


**Fig. S1. DDM element analysis, represents the relative proportion of DDM elements.**


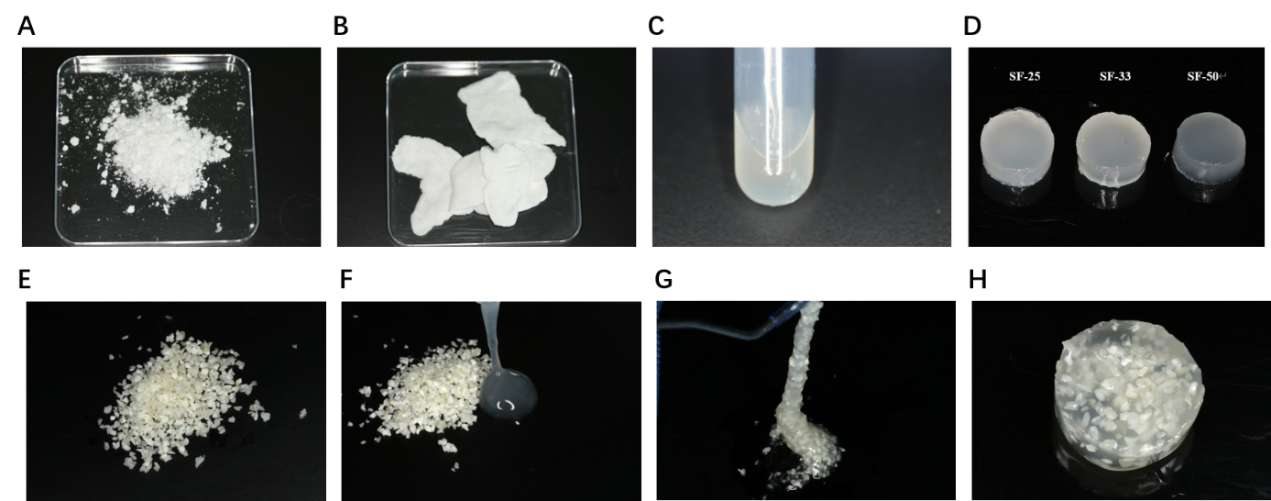


Fig. S2. Production process and performance of SF/HAMA/DDM


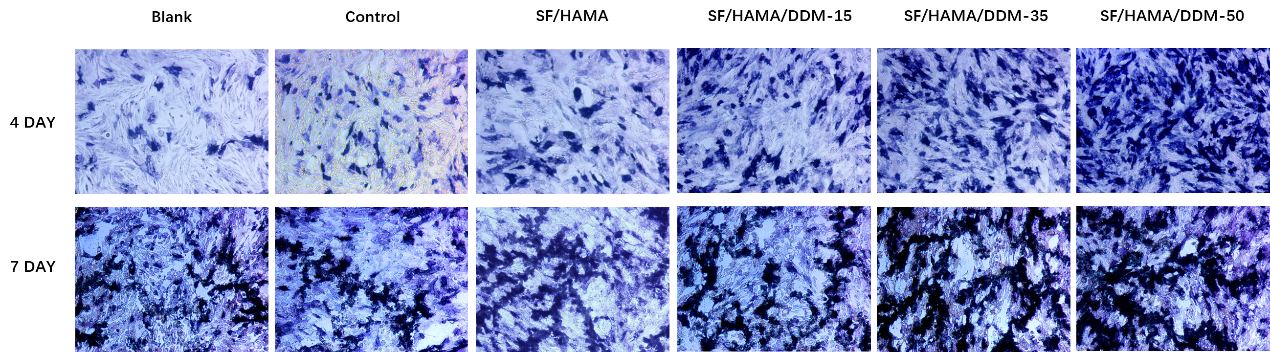


**Fig. S3. ALP staining of MC3T3.**

**
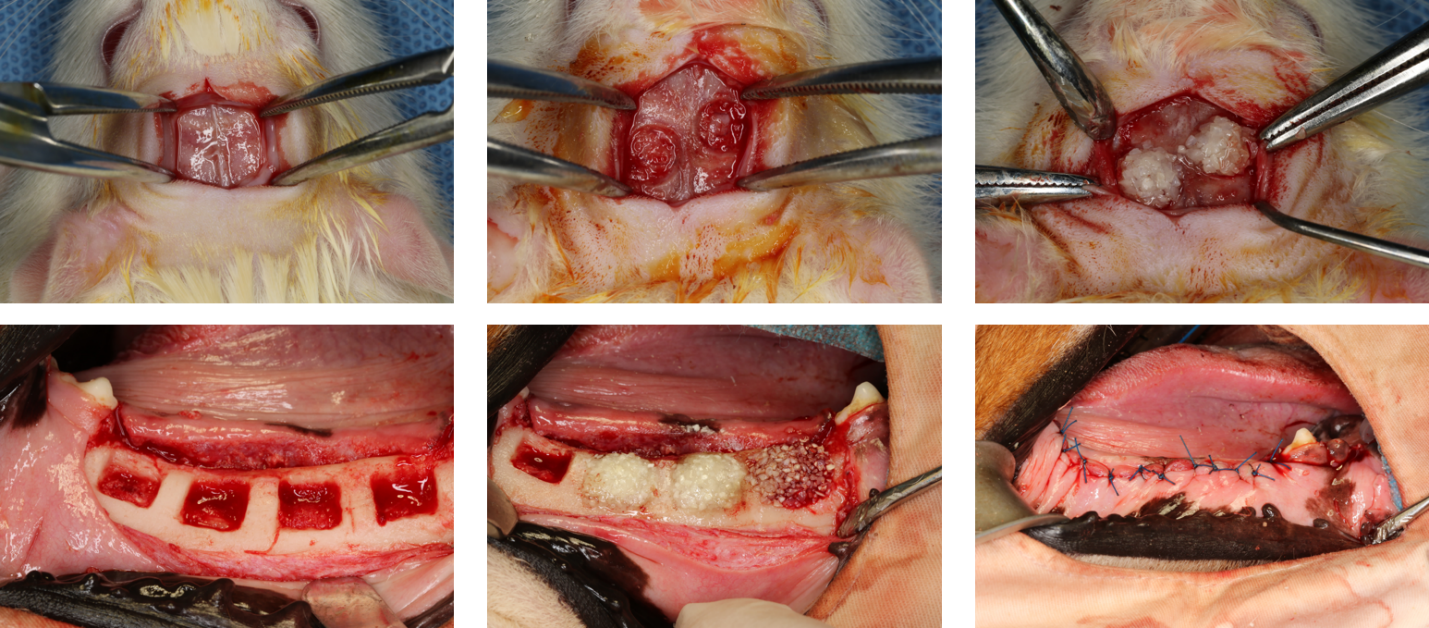
**

**Fig. S4.** **Animal experiments of SF/HAMA/DDM.**

| **Name** | **Direction** | **Sequence (5’ –3’)** |
| --- | --- | --- |
| **ALP** | **Forward** | **CAGGATTACACCAGGAAGCAAGG** |
|  | **Reverse** | **CTGTCCCAAAGCCGTCAATAGC** |
| **RUNX-2** | **Forward** | **AGGCAGTTCCCAAGCATTTCATC** |
|  | **Reverse** | **AGTGAGTGGTGGCGGACATAC** |
| **COL-1** | **Forward** | **GAGGGCCAAGACGAAGACATC** |
|  | **Reverse** | **CAGATCACGTCATCGCACAAC** |
| **OCN** | **Forward** | **GGGCAGCGAGGTAGTGAAGAG** |
|  | **Reverse** | **TCAGCCAACTCGTCACAGTCC** |
| **OPN** | **Forward** | **ATTGGGACAGCCGTGGGAAG** |
|  | **Reverse** | **ATCGGAATGCTCATTGCTCTCATC** |
| **GAPDH** | **Forward** | **AACATCATCCCTGCCTCTACTGG** |
|  | **Reverse** | **GCCTGCTTCACCACCTTCTTG** |

**Table S1. Primers used for real-time RT-PCR**

| Sample Number | Mass(mg) | Peak Area | Adsorption Capacity(ml/g) | Specific Surface Area(m^2^/g) |
| --- | --- | --- | --- | --- |
| 1 | 200.3 | 12536.50 | 0.3086 | 1.0763 |

Table S2. The Test Results of BET Single-point Specific Surface Area
